# When does risk perception predict protection motivation for health threats? A person-by-situation analysis

**DOI:** 10.1371/journal.pone.0191994

**Published:** 2018-03-01

**Authors:** Rebecca A. Ferrer, William M. P. Klein, Aya Avishai, Katelyn Jones, Megan Villegas, Paschal Sheeran

**Affiliations:** 1 Basic Biobehavioral and Psychological Sciences Branch, Behavioral Research Program, National Cancer Institute, Bethesda MD, United States of America; 2 Behavioral Research Program, National Cancer Institute, Bethesda MD, United States of America; 3 Department of Psychology and Neuroscience, University of North Carolina Chapel Hill, Chapel Hill NC, United States of America; University of Saint Andrews, UNITED KINGDOM

## Abstract

Although risk perception is a key concept in many health behavior theories, little research has explicitly tested *when* risk perception predicts motivation to take protective action against a health threat (protection motivation). The present study tackled this question by (a) adopting a multidimensional model of risk perception that comprises deliberative, affective, and experiential components (the TRIRISK model), and (b) taking a person-by-situation approach. We leveraged a highly intensive within-subjects paradigm to test features of the health threat (i.e., perceived severity) and individual differences (e.g., emotion reappraisal) as moderators of the relationship between the three types of risk perception and protection motivation in a within-subjects design. Multi-level modeling of 2968 observations (32 health threats across 94 participants) showed interactions among the TRIRISK components and moderation both by person-level and situational factors. For instance, affective risk perception better predicted protection motivation when deliberative risk perception was high, when the threat was less severe, and among participants who engage less in emotional reappraisal. These findings support the TRIRISK model and offer new insights into when risk perceptions predict protection motivation.

## Introduction

Most individuals would acknowledge that the likelihood they will be diagnosed with a brain tumor is extremely low. However, some of those same individuals might confess to feeling fearful about the possibility of a brain tumor, despite its low odds. Moreover, independent of the perceived likelihood and fear of a brain tumor, individuals may feel vulnerable at a “gut-level”–they could easily imagine being diagnosed with a brain tumor. These three types of risk perception–deliberative, affective, and experiential–are distinct, albeit related, constructs [1]. But which of the three types of risk perception best predicts people’s motivation to protect themselves against health threats? And when, or for whom, are these three different risk perceptions influential? The present study is the first to tackle these questions.

### The TRIRISK model of risk perception and protection motivation

Risk perceptions refer to people’s beliefs and feelings about the possibility of disease or other harms to health, and are accorded a central role in many health behavior theories [2–6] According to these theories, perceived risk is a key predictor of both motivation to take protective action, and subsequent performance of health behaviors geared at alleviating the threat. Despite the conceptual prominence of perceived risk, however, several meta-analyses have observed that risk perception only modestly predicts intentions and health behavior in both correlational [7–9] and experimental tests [10].

One explanation of the modest predictive validity of risk perception is that most tests focus solely on deliberative risk judgments and neglect the potential influence of affective and experiential risk judgments. However, the distinctions among deliberative, affective, and experiential components of risk lie at the heart of the TRIRISK model. *Deliberative* risk perception is a reason-based judgment of probability–the type of risk perception that is most frequently invoked in health behavior theories [2–6] and other models of decision-making [11–14]. *Affective* risk perception refers to the valence (positive-negative) and arousal (high-low) of feelings associated with the threat, and is typically measured by reports of worry, anxiety, or fear [15–16]. The third type is *experiential* risk perception and refers to heuristic risk judgments or “gut-level” feelings of vulnerability to a threat [13,17–20]. Although affective and experiential risk perceptions are often conflated [12,19,21–28], the discriminant validity of all three components (deliberative, affective, and experiential) of risk perception is supported by confirmatory factor analyses. Moreover, there is initial and promising evidence to suggest that all three components are independently associated with protection motivation in relation to cancer [1].

### The present study

The current study had three aims: (1) to determine whether deliberative, affective, and experiential risk perceptions predict protection motivation in relation to a wide a range of health threats, (2) to test whether these different risk perceptions exhibit additive or synergistic relationships in predicting protection motivation, and (3) to examine factors that influence how well each type of risk perception predicts protection motivation. To offer a comprehensive test, we adopted a highly intensive within-subjects paradigm [29] where participants completed measures of deliberative, affective, and experiential risk perceptions and perceived severity in relation to 32 different health threats.

We took a person-by-situation approach and tested both features of the health threat and individual difference variables as moderators of the relation between TRIRISK components and protection motivation. In particular, we assessed perceived severity as a key feature of the health threat, and distinguished between physical severity (how serious would the impact of the threat be for physical health) and psychosocial severity (how much the health threat would interfere with valued social roles) [30]. Several individual differences relevant to moderation of deliberative, affective, and experiential influence were assessed, selected based on their conceptual association between the different types of risk perceptions: need for cognition [31], need for affect [32], faith in intuition [32], cognitive reflection (i.e., reasoned vs. intuitive responding) [33], and dispositions to regulate emotion either by changing how one thinks about the situation (cognitive reappraisal) or by stifling the expression of emotion (suppression) [34]. The individual difference variables were selected based on their relevance to the TRIRISK components. Specifically, high need for cognition and reasoned responding are deliberative traits; reappraisal, suppression, and need for affect are affective traits; and faith in intuition and intuitive responding are experiential traits.

We predicted that the TRIRISK components would be empirically distinct, as evidenced both by intercorrelations less than unity (Hypothesis 1) and that the three would exhibit differential correlations with other variables (Hypothesis 2). Specifically, we predicted that deliberative risk perceptions would be associated with need for cognition and reasoned responding (Hypothesis 2a); affective risk perceptions would be associated with reappraisal, suppression, and need for affect (Hypothesis 2b); and experiential risk perceptions would be associated with faith in intuition and intuitive responding (Hypothesis 2c). Consistent with previous work [1], we also hypothesized that each TRIRISK component would predict unique variance in protection motivation (Hypothesis 3).

We had several hypotheses regarding moderation. Based on previous work, we predicted that deliberative and affective risk perceptions would interact, such that protection motivation would be greatest when deliberative and affective risk perceptions were both high [22,35,36] (Hypothesis 4). We also planned to examine the interactions among experiential risk perceptions and the other two types of risk perception in predicting protection motivation, although we had no specific hypothesis about the nature of the association as no previous work has addressed this issue. Finally, based on the conceptual mapping of risk perception type to respective traits, we predicted that deliberative risk perceptions would more strongly predict motivation when need for cognition and cognitive reflection were high (Hypothesis 5a); affective risk perceptions would better predict motivation when perceived severity, need for affect, and suppression were high, and when cognitive reappraisal was low (Hypothesis 5b); and experiential risk perceptions would most strongly predict motivation when perceived severity and faith in intuition were high.

## Materials and methods

This research was approved by the University of North Carolina at Chapel Hill IRB.

### Participants and procedure

Participants were 94 individuals (51.1% female, *M*_*age*_ = 34.05, *SD* = 11.31). Participant race was White (86.3%), Black (7.4%), Asian (4.2%), and Other (2.1%). Education was college (45.3%), some college (34.7%), high school (19.7%), and less than high school (2.1%). Initially, one-hundred-and-one participants were recruited online using Amazon Mechanical Turk and were paid $5.00 for their participation. This recruitment procedure offers data of equal or higher quality than lab-based samples, and affords relatively greater diversity than student samples and some community samples [37–38].

Based on recommendations for multilevel modeling, we calculated power based on the top level of the multilevel hierarchy (i.e., person-level) [39]. G*Power (http://www.gpower.hhu.de/en.html) indicated that 84 participants would yield 0.90 power to detect associations in a random effects model, assuming a two-tailed test, small-medium effect size (selected conservatively, based on a medium-large effect size in previous research [1]), and three predictors (one risk perception component, one threat characteristic or individual difference, and the interaction between the two). Because multiple regressions were performed (with different combinations of risk perceptions and moderators), we adopted *α* = 0.001 for power calculations (as opposed to the standard 0.05). Power calculations were performed at the participant (rather than observation) level to account for participant-level predictors (individual differences) [40]. Seven participants were excluded from analyses because they failed one or more of three attention check items that were embedded throughout the study [41–42]. The final sample comprised 94 participants.

Participants first rated 32 health threats on the following scales which were presented in a randomized order: Deliberative Risk, Affective Risk, Experiential Risk, Physical Severity, and Psychosocial Severity. Next, participants saw the following measures in a fixed order: Protection motivation in relation to the 32 threats, the Rational Experiential Inventory, the Need for Affect Questionnaire, the Emotion Regulation Questionnaire, and the Cognitive Reflection Test. Finally, participants answered a series of demographic questions. The average time to study completion was 28.39 minutes (*SD* = 10.3, range 10.3–79.4).

### Measures: Perceptions of health threats

Risk perceptions and perceived severity were assessed in relation to 32 health conditions. The 32 conditions were selected from a previous study (see Table 1) [29] and pilot work that indicated that these conditions showed a diverse range of severity. Because we focused specifically on health threats, we replaced “losing $1000 to a con man” with “nosebleed.” We also replaced “starving to death” with “gaining 15+ pounds in weight,” given that weight gain is a more relevant and common threat. The conditions were: sleep-walking; allergy to bananas; nosebleed; head lice; appendicitis; black eye; persistent ear ringing; dislocated finger; addiction to cocaine; syphilis; gaining 15+ pounds in weight; dependency on tranquilizers; pneumonia; cancer from pollution; concussion; tooth needs to be pulled; conjunctivitis; hemorrhoids; strep throat; rash from poison ivy; athlete’s foot; postnasal drip (runny nose); being choked; static shock; high blood pressure; slipped or ruptured disk; ulcer; serious heart attack; gum disease; cataracts; skin cancer; and arthritis.

**Table 1:** Pilot study: Correlations among deliberative, affective, and experiential risk perceptions across threats (T1 and T2).

	Deliberative-Affective		Deliberative-Experiential		Affective- Experiential	
	T1	T2	T1	T2	T1	T2
Cancer (scale)	.308*	.451	.434	.529	.735	.792
Gum disease (single-item)	.404	.462	.586	.596	.714	.600
Weight gain (single-item)	.399	.371	.622	.573	.675	.567
Strep throat (single-item)	.218	.267	.389	.551	.607	.596
Skin cancer (single-item)	.467	.344	.603	.586	.619	.669
Heart attack (single-item)	.499	.438	.605	.557	.753	.702
Cancer (single-item)	.262**	.536	.415	.492	.637	.591

Note: all correlations p < .001 unless otherwise noted

* *p* = .001

** *p* = .005

Given the considerable burden of rating three types of risk perceptions, two types of perceived severity, and protection motivation in relation to 32 threats, single-item measures were used. Deliberative risk perceptions were assessed by asking: “How likely is it that you will get these conditions at some point in the future?” (1 = *Unlikely–* 7 = *Likely*). Affective risk perceptions were indexed by the item: “How fearful are you of getting these conditions in the future?” (1 = *Not at all–* 7 = *Extremely*). The experiential risk perception item was: “I feel very vulnerable to [threat]” (1 = *Strongly disagree–* 7 = *Strongly agree*). These items were selected based on the strength of their factor loadings (onto the three overarching constructs) in previous research validating the full scale [1]. We conducted a separate study (*N* = 116) to establish the reliability of the single-item measures of the risk components (full details of the study are presented in the Supplementary Materials). Findings showed that across six different threats, the mean test-retest reliabilities for deliberative, affective, and experiential risk perceptions over two weeks were *r* = .71, .66, and .63, respectively (*SE* = .04, .04, and .03) (Table 1). Thus, our single-item indices of TRIRISK components exhibited satisfactory reliability.

Physical and psychosocial severities were assessed by “How serious would these conditions be in physical/medical terms (e.g., symptoms, pain, treatment, hospitalization)?” (0 *= Innocuous*, *no harm at all–* 10 = *Extremely devastating*); and “How serious would these conditions be in personal terms (e.g., your feelings about yourself, your relationship with others)?” (0 *= Innocuous*, *no harm at all–* 10 = *Extremely devastating*), respectively.

Protection motivation was assessed with a single item [29]: “Imagine that you were given the power to make certain that each condition would never happen to you. But this power is limited; you cannot eliminate all of the risks. Which ones would you give the highest priority? Please note that you should not choose more than four conditions per priority level.” (1 = *Lowest priority–* 10 = *Top priority)*. Given the complexity of the study, it was not feasible to collect behavioral data for the 32 hazards. We did, however, undertake a separate study with MTurk participants (*N* = 40) to validate our measure of protection motivation. Protection motivation was measured with the item used in the main study, intention was assessed using the item “How much do you intend to protect yourself from each of the following conditions? (7-point scale; no intention-very strong intention), and behavior was indexed by the item “How often do you behave so as to protect yourself from each of the following conditions? (7-point scale; never-every time). Protection motivation, intentions, and behavior were assessed in relation to 12 of the behaviors used in the main study, and scales proved reliable (alphas = .89, .95, and .94, respectively). Findings showed that protection motivation was significantly correlated with both intentions (*r* = .74, *p* < .001) and behavior (*r* = .47, *p* = .002). These findings suggest that our measure of protection motivation is valid. Moreover, previous research documents that protection motivation, while not a proxy for behavior, is a reliable precursor to behavior [43–44]

Need for cognition and faith in intuition were assessed with the short version of the Rational Experiential Inventory (10 items) [32]. There were five need for cognition items (α = .90) and five faith in intuition items (α = .88), with responses on a 5-point scale (1 = *Completely false–* 5 = *Completely true*). An example of a need for cognition item is: “I prefer complex to simple problems.” An example of a faith in intuition item is: “I trust my initial feelings about people.” The ten-item version of the Need for Affect Questionnaire [30] was used and rated on a seven-point scale from -3 to 3 (*Strongly disagree–Strongly agree*). There were five avoidance items (α = .85) and five approach items (α = .89). An example of an approach item is: “I think it is important to explore my feelings.” An example of an avoidance item is: “I do not know how to handle my emotions, so I avoid them.” Reappraisal and suppression were assessed with the ten-item Emotion Regulation Questionnaire [34] on a scale from 1 to 7 (*Strongly disagree*–*Strongly agree*). There were six reappraisal items (α = .93) and four suppression items (α = .85). An example of a reappraisal item is: “When I want to feel less negative emotion, I change the way I’m thinking about the situation.” An example of a suppression item is: “I keep my emotions to myself.” The order of these items was not randomized because the first and third items defined positive and negative emotion, respectively.

The 3-item Cognitive Reflection Task [31] was used and open-ended responses were allowed. The items were: (1) A bat and a ball cost $1.10 in total. The bat costs $1.00 more than the ball. How much does the ball cost (in cents)? (2) If it takes five machines five minutes to make five widgets, how long would it take 100 machines to make 100 widgets (in minutes)? (3) In a lake, there is a patch of lily pads. Every day, the patch doubles in size. If it takes 48 days for the patch to cover the entire lake, how long would it take for the patch to cover half of the lake (in days)? Responses were coded as incorrect (*0*) or correct (*1*) and summed (α = .76). Despite its proliferation, the mean for this 3-point scale was 1.84 (*SD* = 1.19), demonstrating variability in correct responses to the scale.

### Demographics

Participants responded to items regarding their gender, age, education level, and race/ethnicity. Participants also completed items about subjective socioeconomic status using the following item (response options 1 through 10): “Think of this ladder as representing where people stand in the United States. At the top of the ladder (rung 10) are the people who are the best off—those who have the most money, the most education and the most respected jobs. At the bottom of the ladder (rung 1) are the people who are the worst off—those who have the least money, least education, and the least respected jobs or no job. The higher up you are on this ladder, the closer you are to the people at the very top; the lower you are, the closer you are to people at the very bottom. Where would you place yourself on this ladder? Click the number of the rung where you think you stand at this time in your life, relative to other people in the United States.^”^ [45]. Mean score on this item was 5.31 (*SD* = 1.91). They also completed a categorical item assessing household income, starting at “less than $5,000” and going up in $5,000 intervals to “$175,000 or more.” This was treated continuously (as a scale from 1–19) and the mean score was 9.85 (corresponding to an income of $30,000–35,000), *SD* = 4.41 (corresponding to a SD of approximately $25,000).

### Analyses

To examine the hypothesized tripartite structure of risk perceptions, we examined correlations among the three risk perceptions and other variables (threat characteristics and individual differences). Fisher’s *r-*to-*z* transformations were used to test whether correlations were significantly different from one another, and discriminant validity was assessed by whether each type of risk perception had different correlations with other variables [46].

To examine what factors modify the strength of association between each type of risk perception and protection motivation, we conducted mixed model, random-effects regressions with maximum likelihood estimation in SAS 9.3 to account for the within-subjects repeated measures design, where the threats were nested within subject. The final analyses involved 2968 observations–a number that accounts for listwise deletion of missing data (at the observation, rather than participant, level) from the total 3040 observations possible in the design (94 participants by 32 threats). All predictors were centered prior to the computation of interaction terms. All analyses controlled for gender, age, education level, subjective socioeconomic status, yearly household income, and race/ethnicity.

We computed two sets of models: 1) regressing protection motivation on the three risk perception components (deliberative, affective, and experiential risk perceptions) and interactions among these components; and 2) regressing protection motivation on the three risk components, physical and psychosocial severity of the threats, and individual difference variables (emotion regulation, cognitive reflection, need for cognition, need for affect, and faith in intuition; these variables were at the participant- rather than threat-level, and were represented in each line of data), as well as interactions among the three risk perceptions and severity and individual differences. Within each set of models, analyses involved the following steps: 1) regressing the outcome on each interaction term (and its lower-order main effects terms); 2) regressing the outcome on significant interaction terms (and their lower-order main effects terms) in a comprehensive model; 3) trimming non-significant interaction terms from the comprehensive model and conducting a final comprehensive model; and 4) regressing motivation on all lower-order main effects terms (absent interactions, to generate meaningful estimates of main effects). Main effects reported are from comprehensive models generated in Step 4; interaction terms reported are from comprehensive models generated in Step 3. Simple slopes [47] were calculated for continuous interactions using one standard deviation above and below the mean as critical values. All study data are available for download (S1 File).

## Results and discussion

### Discriminant validity of the TRIRISK model of risk perception

**Fig 1:**
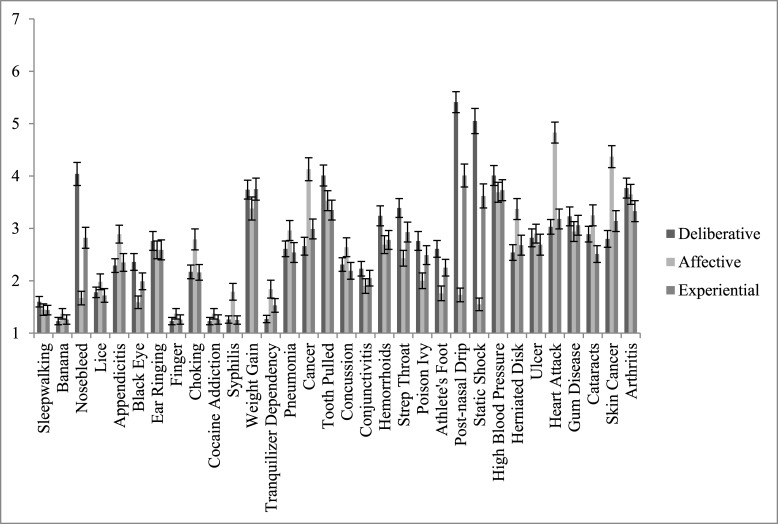
Distributions of TRIRISK components by threat.

The distribution of deliberative, affective, and experiential risk perceptions for each health threat is presented in Fig 1, and indicates that there was substantial variability in participants’ risk perceptions across the different threats. The means, standard deviations and intercorrelations for study variables are presented in Table 2. Two lines of evidence supported the discriminant validity of TRIRISK components. Supporting Hypothesis 1, correlations among deliberative, affective, and experiential risk perceptions were considerably less than unity (*r*s = .29 to .66; see Table 2). In partial support of Hypothesis 2, the three types of risk perception exhibited different correlations with other variables, with mixed support for predictions. Deliberative risk perceptions were not associated with need for cognition or cognitive reflection (Hypothesis 2a). However, they were *negatively* correlated with faith in intuition (*r* = -.11, *p* < .01), which is consistent with the idea that those who process more intuitively might rely less on deliberation. As predicted, affective risk perceptions were associated with reappraisal and suppression (*r*s = .08 and -.15, respectively, *p*s < .01) and with physical and psychosocial severity (*r*s = .39 and .37, respectively, *p*s < .01), and was associated with the approach (*r* = 0.20), but not the avoidance, component of need for affect (Hypothesis 2b). As predicted, experiential risk perceptions were correlated with cognitive reflection scores, such that higher experiential risk perceptions were observed for more intuitive participants (*r* = -.07, *p* < .01); however, experiential risk perception was not associated with scores on the faith in intuition scale (Hypothesis 2c).

**Table 2:** Means, standard deviations and correlations among study variables.

	1		2		3		4		5		6		7		8		9		10		11		12		13
	*r*	*p*	*r*	*p*	*r*	*p*	*r*	*p*	*r*	*p*	*r*	*p*	*r*	*p*	*r*	*p*	*r*	*p*	*r*	*p*	*r*	*p*	*r*	*p*	
1 Deliberative RPs	1																								
2 Affective RPs	.29	< .001	1																						
3 Experiential RPs	.66	< .001	.44	< .001	1																				
4 Physical Severity	-.12	< .001	.39	< .001	.05	.012	1																		
5 Psycho. Severity	-.09	< .001	.37	< .001	.06	.001	.66	< .001	1																
6 Need for Cogn.	-.03	.058	-.01	.751	-.08	< .001	-.04	.031	-.01	.689	1														
7 Faith in Intuit.	-.11	< .001	.01	.551	-.04	.032	-.05	.012	-.03	.064	.04	.018	1												
8 NfA Avoidance	.01	.555	.04	.055	.05	.008	.01	.691	.01	.827	-.39	< .001	-.17	< .001	1										
9 NfA Approach	.04	.024	.20	< .001	.09	< .001	-.02	.310	-.02	.328	-.30	< .001	.04	.014	-.48	< .001	1								
10 Reappraisal	-.04	.022	.08	< .001	-.06	< .001	.07	< .001	.06	< .001	.24	< .001	.21	< .001	-.35	< .001	.14	< .001	1						
11 Suppression	-.01	.626	-.15	< .001	.01	.512	-.04	.037	-.02	.238	-.20	< .001	-.08	< .001	.52	< .001	.50	< .001	-.17	< .001	1				
12 Cognitive Refl.	-.03	.130	-.02	.348	-.07	< .001	-.04	.021	-.03	.097	.16	< .001	-.17	< .001	.08	< .001	-.14	< .001	-.03	.061	.20	< .001	1		
13 Motivation	-.04	.025	.44	< .001	.13	< .001	.66	< .001	.54	< .001	-.01	.699	-.03	.110	.01	.642	.01	.500	.01	.838	-.02	.232	.01	.783	
*M*	2.80		2.60		2.55		5.62		4.20		3.79		3.73		-1.14		.85		5.28		3.55		1.84		5.44
*SD*	1.85		1.88		1.84		3.24		3.36		.98		.77		1.35		1.32		1.11		1.42		1.19		3.04
Test-retest*	.74		.92		.85																				

*Test-retest data are drawn from a separate pilot study reported in the supplementary materials I.

Comparisons of these correlations indicated that deliberative and affective risk perceptions differed in the degree to which they were correlated with faith in intuition (*z* = 2.71, *p* = .007); reappraisal (*z* = 4.63, *p* < .001); and suppression (*z* = 5.43, *p* < .001). Deliberative and experiential risk perceptions differed in the degree to which they were correlated with physical severity (*z* = 6.57, *p* < .001), psychosocial severity (*z* = 5.79, *p* < .001), and cognitive reflection (*z* = 6.57, *p* < .001). Affective and experiential risk perceptions differed in the degree to which they were correlated with physical severity (*z* = 13.93, *p* < .001), psychosocial severity (*z* = 12.64, *p* < .001), need for affect (approach) (*z* = 4.33, *p* < .001), reappraisal (*z* = 5.40, *p* < .001), and suppression (*z* = 6.20, *p* < .001).

### Main effects and interactions among TRIRISK components in predicting protection motivation

Table 3 presents the main effects and interactions for deliberative, affective, and experiential risk perceptions from the multilevel model predicting protection motivation (Hypotheses 3 and 4). All three types of risk perception predicted protection motivation, with affective risk perception being the most powerful predictor (*d* = 1.14). Whereas the associations between protection motivation and affective and experiential risk perceptions were both positive, the association was negative in the case of deliberative risk perception. Negative cross-sectional associations between deliberative risk perceptions and intentions are not uncommon [1,48], as participants who are motivated to protect themselves against threat may (accurately) assess their risk as lower.

**Table 3:** Main effects and interactions among three risk perceptions in predicting protection motivation (*n* = 2968).

	Main Effects				Interactions with Deliberative			
	*B*	*95% CI*	*p*	*d*	*B*	*95% CI*	*p*	*d*
Deliberative Risk Perceptions	-0.40	-0.47, -0.33	**< .001**	-0.41				
Affective Risk Perceptions	0.95	0.89, 1.01	**< .001**	1.14	0.05	0.02, 0.08	**< .001**	0.13
Experiential Risk Perceptions	0.11	0.03, 0.19	**.007**	0.10	-0.04	-0.07, -0.01	**.006**	-0.10

There were two significant interactions among the three risk perception types in predicting protection motivation. Deliberative and affective risk perceptions interacted with one another (*B* = 0.05, *p* < .001, *d* = 0.13) such that affective risk perceptions better predicted protection motivation when deliberative risk perception was high (*B* = 1.00, *p* < .001, *d* = 1.01) as compared to low (*B* = 0.82 *p* < .001, *d* = 0.65). The interaction between deliberative and experiential risk perceptions was also significant (*B* = -0.04, *p* < .001, *d* = 0.10). Experiential risk perception predicted protection motivation when deliberative risk perception was low (*B* = 0.22, *p* < .001, *d* = 0.14), but not when deliberative risk perception was high (*B* = 0.07, *p* = .093, *d* = 0.06).

### Interactions between TRIRISK components and person-level and situational factors

Table 4 presents the findings from multilevel models predicting protection motivation from the three risk perceptions, person and situational factors, and their interactions. There were no significant interactions between psychosocial severity or emotion suppression and any of the three risk perceptions in predicting protection motivation, so these variables were excluded from the final analyses.

**Table 4:** Main effects and interactions among three risk perceptions, health threat characteristics, and individual differences (*n* of observations = 2968).

	Main Effects				Interaction Terms											
					Deliberative				Affective				Experiential			
	*B*	*95% CI*	*p*	*d*	*B*	*95% CI*	*p*	*d*	*B*	*95% CI*	*p*	*d*	*B*	*95% CI*	*p*	*d*
Deliberative Risk	-0.11	-0.17,-0.05	**< .001**	-0.14												
Affective Risk	0.51	0.45,0.56	**< .001**	0.63												
Experiential Risk	0.09	0.03,0.16	**.001**	0.10												
Need for Cognition	0.05	-0.24,0.33	.733	0.07	0.05	0.01,0.09	**.032**	0.08	-		-		-		-	
Reappraisal	-0.21	-0.46,0.03	.092	0.39	-		-		-0.06	-0.10,-0.02	**.002**	-0.11	-		-	
Physical Severity	0.55	0.51,0.57	**< .001**	1.45	-		-		-0.03	-0.04,-0.02	**< .001**	-0.17	-		-	
Cognitive Reflection	0.07	-0.14,0.33	.562	0.17	-		-		-		-		-0.05	-0.08,-0.01	**.009**	-0.10

Consistent with our proposition that deliberative, affective, and experiential risk perceptions are distinct constructs, different factors moderated the associations between the three types of risk perception and protection motivation. As predicted (Hypothesis 5a), need for cognition moderated the influence of deliberative risk perception (*B* = 0.05, *p* = .032, *d* = 0.08), such that deliberative risk perception was negatively related to protection motivation observed among participants designated low in need for cognition (*B* = -0.16, *p* < .001, *d* = -0.16), but was not associated with protection motivation among participants high in need for cognition (*B* = -0.06, *p* = .073, *d* = -0.06). Also as predicted (Hypothesis 5b), use of reappraisal as an emotion regulation strategy (*B* = -0.06, *p* = .002, *d* = -0.11) and physical severity (*B* = -0.03, *p* < .001, *d* = -0.17) both moderated associations for affective risk perception. Affective risk perceptions better predicted protection motivation when reappraisal scores were low (*B* = 0.58, *p* < .001, *d* = 0.54) as compared to high (*B* = 0.44, *p* < .001, *d* = 0.45), and when threats were perceived as less severe (*B* = 0.56, *p* < .001, *d* = 0.59) than more severe (*B* = 0.44, *p* < .001, *d* = 0.45). Finally, cognitive reflection (*B* = -0.05, *p* = .009, *d* = -0.10) moderated the strength of the associations observed for experiential risk perception, consistent with Hypothesis 5c. In particular, experiential risk perceptions predicted protection motivation when cognitive reflection was low (*B* = 0.15, *p* < .001, *d* = 0.15), but not when cognitive reflection was high (*B* = 0.03, *p* = .379, *d* = 0.03).

## Conclusion

Zanna and Fazio [49] delineated three generations of research on construct-behavior relations that can also be used to characterize the study of risk perception, a key construct in health behavior theories. The research question addressed in the first generation is “*Does* risk perception predict protection motivation?” The second-generation question is “*When* does risk perception predict protection motivation?” and the third-generation question is “*How* does risk perception predict protection motivation?” The present research makes valuable progress in addressing the first-generation question and offers an initial but comprehensive test of the second-generation research question.

Whereas previous research indicated that risk perceptions sometimes fail to predict protection motivation, and exhibit sample-weighted effect sizes of small magnitude [9–10], the present study showed that prediction is enhanced by characterizing risk perceptions in terms of three distinct, albeit related, components: deliberative, affective, and experiential (the TRIRISK model). The tripartite structure of risk perceptions was supported here by findings showing that the three components were moderately correlated. It is notable that affective and experiential risk perceptions were *less* related to one another than were experiential and deliberative risk perceptions [1]. The three types of risk perceptions also exhibited different patterns of association with other variables, demonstrating the discriminant validity of these risk components. All three risk perceptions were associated with protection motivation and explained additional variance beyond that engendered by each component on its own. Affective risk perceptions emerged as the strongest predictor, a finding consistent with previous work [1,24–25,50].

Significant interactions were observed between deliberative risk perception and both affective and experiential risk perceptions that explained significant increments in the variance of protection motivation. Affective risk perception better predicted protection motivation when deliberative risk perception was high, a finding consistent with evidence regarding diabetes risk perceptions and protection motivation [35] (but see also [22,36]). Conversely, experiential risk perception predicted protection motivation when deliberative risk perception was low, but not high. Thus, when the multiple components of risk perception and interactions among those components are taken into consideration, it appears that risk perception *does* predict protection motivation.

Little systematic research has tackled the second-generation question of *when* risk perception predicts protection motivation. Here, we took a person-by-situation approach and tested whether features of the health threat and individual difference variables both moderate the relationship between risk perceptions and protection motivation. Although psychosocial severity did not interact with any of the three types of risk perception, the perceived physical severity of the threat moderated the association for affective risk perception such that affective risk perception was more strongly associated with protection motivation when the health threat was seen as less serious. It appears that fear becomes less motivating as the potential health threat grows more serious. This finding would seem to be out of line with studies of fear appeals, which characteristically find that fear better predicts intention when the threat is more versus less severe [3,51]. However, it is possible that there is a restricted range of affective risk perception and perceived severity ratings in fear appeals research (i.e., researchers generally study issues where it is possible to increase fear or perceived severity [29]). The 32 health threats examined in the present study, on the other hand, exhibited considerable variability in ratings of severity and affective risk, which may have enabled us to detect this interaction. Further research that varies the range of health threats in terms of their severity and affective risk would be valuable to test this hypothesis.

Several individual differences moderated relations between TRIRISK components and protection motivation. Of interest, we observed that deliberative, affective, and experiential risk perceptions each interacted with different individual difference variables. Specifically, need for cognition moderated deliberative risk perceptions, emotional reappraisal moderated affective risk perceptions, and cognitive reflection moderated experiential risk perceptions. Findings were generally in line with expectations. The more participants used reappraisal to regulate their emotions, the weaker was the association between affective risk perception and protection motivation. Similarly, when participants’ responses on the cognitive reflection test were more intuitive, experiential risk perception was associated with protection motivation, but when responses were more reasoned, experiential risk perception was no longer a significant predictor. We also found that need for cognition moderated the negative association between deliberative risk perception and perception motivation documented here (and elsewhere [1,43,52]); a reliable, negative association was only observed for participants with low scores on need for cognition. In sum, the present research offers new answers to the question “When does risk perception predict protection motivation?” Situational (physical severity) and person-level factors (need for cognition, reappraisal, and cognitive reflection) both influence how well risk perceptions are translated into motivation to protect oneself from harm.

As with any new program of research, this study has limitations. The design was cross-sectional and the study focused on protection motivation rather than protective behavior. These features of the research are justifiable in this, first test of TRIRISK components in relation to a wide range of (a) health threats, and (b) moderator variables. However, we acknowledge that experimental tests that manipulate deliberative, affective, and experiential risk perceptions and perceived severity are a crucial next step [53], as is measurement of behavioral outcomes. We examined only one feature of the health threat (perceived severity) in the present study, and other features warrant investigation (e.g., proximity, visibility). Other individual difference variables, not examined here, also warrant study (e.g., need for closure). More complex analyses that explore 3-way interactions among risk components (deliberative, affective, and experiential), features of the threat, and individual differences were beyond the scope of the present paper and constitute another important avenue for future research. The generalizability of the present findings also remains to be determined, despite research suggesting mTurk samples provide high quality data similar to estimates in other samples [37–38], and the hypotheses examined here also need to be tested among more socioeconomically and racially diverse samples.

Notwithstanding these limitations, the present study deployed a highly intensive but under-utilized within-participants paradigm [29] that offered new insights into when risk perceptions are associated with protection motivation. Our findings suggest that affective risk perceptions will be the most powerful predictor of whether or not the individuals in the opening paragraph elect to protect themselves against the possibility of a brain tumor. The person’s gut feelings (experiential risk perceptions) and probability judgments (deliberative risk perceptions) will also be important. Fear of a possible brain tumor (affective risk perception) will be less influential if this condition is seen as deeply serious and if the individuals characteristically use reappraisal to regulate their feelings. Deliberative risk perception will be less influential if people are high in need for cognition, whereas if they receive low scores on the cognitive reflection test, then people are more likely to ‘go with their gut.’ In sum, the present study offers a novel empirical analysis that supports the distinctiveness of three types of risk perception (deliberative, affective, and experiential) and indicates how person-level and situational factors influence the predictive validity of these risk components. These findings warrant further tests of the different ways that people construe risk and the factors that determine when risk perceptions relate to efforts to reduce risk.

## Supporting information

S1 File Study data file. (SAS7BDAT)

S2 File Supporting information. (DOCX)
