# Supplementary material for: When does risk perception predict protection motivation for health threats? A person-by-situation analysis
Source: PLoS One. 2018 Mar 1;13(3):e0191994. doi: 10.1371/journal.pone.0191994 (PMC5832213; doi:10.1371/journal.pone.0191994)
Supplement: S2 File — (DOCX) [file pone.0191994.s002.docx]

**Supporting Information**

Because of the intensive multilevel design that required participants to assess TRIRISK components and threat features for 32 health threats, single items were used to assess these constructs. We conducted a pilot study to examine the test-retest reliability of the previously validated TRIRISK scale,^1^ and compared it to test-retest reliability of the single-item measures across a subset of five specific threats.

**Pilot Test Methods**

*Participants and Procedure.* One-hundred-and-fifty participants were recruited online using Prolific Academic and were paid $1.12 for their participation in both waves of the study. The final sample of participants providing data at both waves included 116 individuals (55.2% women; *M*-age = 35.32, *SD* = 12.57, 86.2% White, 3.4% Black, 6.0% Asian, 4.4% Other; 4.3% less than high school, 23.4% high school degree; 23.3% some college; 49.2% college degree).

Participants first completed the full TRIRISK scale regarding their perceived risk for cancer.^1^ They then completed one item each to assess each TRIRISK component for five threats (gum disease, weight gain, strep throat, skin cancer, heart disease). The single-item measures for each TRIRISK component were selected based on their loadings on scales that were validated across five samples and in relation to three target diseases.^28^ Deliberative risk perceptions were assessed by asking: “How likely is it that you will get these conditions at some point in the future?” (1=*Unlikely –* 7=*Likely*). Affective risk perceptions were indexed by the item: “How fearful are you of getting these conditions in the future?” (1=*Not at all –* 7=*Extremely*). The experiential risk perception item was: “I feel very vulnerable to [threat]” (1=*Strongly disagree –* 7=*Strongly agree*). Participants also responded to items regarding their gender, age, education level, yearly household income, and race/ethnicity. Participants were re-contacted 2 weeks later to provide test-retest data for these scales and items.

*Analyses.* All analyses were conducted in SPSS v21. To examine test-retest reliability, we calculated correlations between T1 and corresponding T2 assessments, for both the full scale and single-item assessments (including the single items in the cancer scale that corresponded to the single-item scales for the other threats).

**Pilot Test Results and Discussion**

Test-retest reliability statistics were high, and are presented in Table S1. Findings indicated good test-retest reliability for the multi-item TRIRISK scales (all *r* ≥ .74). Test-retest reliabilities for deliberative risk perception (range = .56 to .82. *M =* .71), affective risk perception (range = .54 to .79. *M =* .66), and experiential risk perception (range = .55 to .73. *M =* .63) were all acceptable. The correlations between the single item measure and the full scale for cancer risk perceptions were high (deliberative *r* = .739, affective *r* = .923, experiential *r* = .846) which suggests that the single-item assessments are likely to be capturing the same construct structure as the full scale. In sum, the present suggest that the strategy of using single items to index the TRIRISK components – in order to reduce participant burden – was legitimate.

**S1 Table.** Test-retest reliability of Deliberative, Affective, and Experiential Risk Perceptions

(scale and single-items)

|  | *r* | *p* |
| --- | --- | --- |
| Deliberative Risk Perception scale (cancer) | .741 | <.001 |
| Affective Risk Perception scale (cancer) | .833 | <.001 |
| Experiential Risk Perception scale (cancer) | .746 | <.001 |
| Deliberative Risk Perception single-item (gum disease) | .818 | <.001 |
| Deliberative Risk Perception single-item (weight gain) | .746 | <.001 |
| Deliberative Risk Perception single-item (strep throat) | .557 | <.001 |
| Deliberative Risk Perception single-item (skin cancer) | .755 | <.001 |
| Deliberative Risk Perception single-item (heart attack) | .750 | <.001 |
| Deliberative Risk Perception single-item (cancer) | .643 | <.001 |
| Affective Risk Perception single-item (gum disease) | .538 | <.001 |
| Affective Risk Perception single-item (weight gain) | .621 | <.001 |
| Affective Risk Perception single-item (strep throat) | .561 | <.001 |
| Affective Risk Perception single-item (skin cancer) | .737 | <.001 |
| Affective Risk Perception single-item (heart attack) | .740 | <.001 |
| Affective Risk Perception single-item (cancer) | .789 | <.001 |
| Experiential Risk Perception single-item (gum disease) | .663 | <.001 |
| Experiential Risk Perception single-item (weight gain) | .552 | <.001 |
| Experiential Risk Perception single-item (strep throat) | .633 | <.001 |
| Experiential Risk Perception single-item (skin cancer) | .606 | <.001 |
| Experiential Risk Perception single-item (heart attack) | .567 | <.001 |
| Experiential Risk Perception single-item (cancer) | .732 | <.001 |

**S2 Table.** Interaction terms from regressions (each interaction from an independent regression)

|  | Interaction Terms | | | | | |
| --- | --- | --- | --- | --- | --- | --- |
|  | Deliberative | | Affective | | Experiential | |
|  | *B* | *p* | *B* | *p* | *B* | *p* |
| Deliberative Risk Perceptions | - | - | **0.04** | **.003** | **-0.10** | **<.001** |
| Affective Risk Perceptions | - | - | - | - | 0.02 | .256 |
| Trust in Intuition | 0.04 | .306 | -0.05 | .203 | 0.01 | .718 |
| Need for Cognition | **.08** | **.016** | 0.02 | .491 | 0.05 | .067 |
| Need for Affect: Approach | -0.01 | .768 | 0.01 | .748 | -0.01 | .942 |
| Need for Affect: Avoidance | -0.01 | .792 | -0.03 | .109 | 0.04 | .122 |
| Reappraisal | -0.03 | .369 | **-0.05** | **.045** | 0.01 | .771 |
| Suppression | -0.03 | .157 | 0.01 | .874 | -0.03 | .159 |
| Physical Severity | **0.03** | **<.001** | **-0.03** | **<.001** | 0.01 | .293 |
| Psychosocial Severity | **0.06** | **<.001** | **-0.02** | **.002** | **0.05** | **<.001** |
| Cognitive Reflection Task | **-1.08** | **.001** | 0.01 | .567 | **-0.12** | **<.001** |
